# Editorial: Novel insights in RNA modifications: From basic to translational research

**DOI:** 10.3389/fcell.2023.1155993

**Published:** 2023-02-22

**Authors:** Huilin Huang, Chengqi Yi, Pengxu Qian, Hengyou Weng, Jianjun Chen

**Affiliations:** ^1^ State Key Laboratory of Oncology in South China, Collaborative Innovation Center for Cancer Medicine, Sun Yat-sen University Cancer Center, Guangzhou, China; ^2^ State Key Laboratory of Protein and Plant Gene Research, School of Life Sciences, Peking University, Beijing, China; ^3^ Peking-Tsinghua Center for Life Sciences, Peking University, Beijing, China; ^4^ Department of Chemical Biology, Synthetic and Functional Biomolecules Center, College of Chemistry and Molecular Engineering, Peking University, Beijing, China; ^5^ Center of Stem Cell and Regenerative Medicine, and Bone Marrow Transplantation Center of the First Affiliated Hospital, Zhejiang University School of Medicine, Hangzhou, China; ^6^ Liangzhu Laboratory, Zhejiang Engineering Laboratory for Stem Cell and Immunotherapy, Zhejiang University Medical Center, Institute of Hematology, Zhejiang University, Hangzhou, China; ^7^ Guangzhou Laboratory, Guangzhou, China; ^8^ State Key Laboratory of Respiratory Diseases, The First Affiliated Hospital, Guangzhou Medical University, Guangzhou, China; ^9^ Department of Systems Biology, Beckman Research Institute of City of Hope, Monrovia, CA, United States; ^10^ Gehr Family Center for Leukemia Research, City of Hope Comprehensive Cancer Center, Duarte, CA, United States

**Keywords:** M^6^A, epitranscriptome, RNA editing, development, cancer, biomarker, RNA vaccine

## Introduction

Chemical modifications add to the diversity of biological macromolecules (e.g., DNA, RNA, and protein) and expand their molecular functions, and the aberrance of such modifications are found as one of the major causes of human diseases and aging (Flavahan et al., 2017; Cavalli and Heard, 2019; Yang et al., 2023). Due to the prominent roles of RNA modifications in human diseases such as cancer and the promise of targeting dysregulated RNA modification machineries in translational medicine (Huang et al., 2020a), the study of RNA modifications (e.g., m^6^A, m^1^A, m^5^C, m^6^Am, pseudouridine, and A-to-I editing) represents the new Frontier in the epigenetics field.

In this Research Topic on *Novel Insights in RNA Modifications: from Basic to Translational Research*, we aim to publish innovative research from basic science to translational research on RNA modifications. A total of 18 articles were included in this Research Topic, covering the novel methods to detect or modulate RNA modifications, the functions and mechanisms of RNA modifications in physiological processes (e.g., normal cellular functions, skeletal myogenesis and fetal development) and during pathogenesis (e.g., cancer, cadiomyopathy, lupus nephritis, inflammatory bowel disease and liver fibrosis), and the application of RNA modification in disease diagnostics and therapeutics. We summarize and discuss the main findings of these studies in this editorial.

## Methodologies for measuring or modulating RNA modifications

The development of reliable m^6^A profiling methods, such as m^6^A-seq or MeRIP-seq, a method that uses antibodies to immunoprecipitate methylated RNAs for subsequent sequencing, greatly promotes our understanding of m^6^A. Recently developed antibody-free techniques, such as DART-seq (Meyer, 2019), m^6^A-SAC-seq (Hu et al., 2022) and GLORI (Liu et al., 2022), exhibit advantages over antibody-based methods, including requirement of less input RNA and eliminated/reduced cross-reactivity to other modifications, such as DNA 6 mA and RNA m^6^Am. In this Research Topic, Zhu et al. developed an improved version of *in vitro* DART-seq, which optimizes the APO1-YTH protein to achieve enhanced m^6^A recognition and allow for m^6^A mapping in any sample of interest using nanogram amounts of total RNA.

Programmable RNA modification is another powerful method for RNA modification study. Hundreds of m^6^A sites are often reprogrammed during physiological and pathological processes, making it difficult to dissect the phenotypic outcomes from a single m^6^A site. The advent of new CRISPR tools allow scientists to install or remove m^6^A modification at specific loci, showing promise in revealing the physiological or pathological effects of individual m^6^A mark, especially *in vivo*. In the review article, Lo et al. summarized recent findings on RNA editing and programmable RNA modification with CRISPR, base editors and non-CRISPR related tools, highlighting their future applications for basic and clinical research.

## The functions and mechanisms of RNA methylations in physiological processes

Utilizing the m^6^A sequencing techniques, the profiling of m^6^A (also known as “epitranscriptome”) under various physiological context can be readily characterized, offering an opportunity for revealing the roles of m^6^A during these processes. Xie et al. characterized the expression and m^6^A methylation patterns of lncRNAs in mouse myoblasts and differentiated myotubes, uncovering a METTL3/m^6^A/Brip1os/Tbx2 Axis and the potential role of m^6^A on the temporal expression regulation of lncRNAs in skeletal myogenesis. Xiao et al. reported that maternal microbiome affects the m^6^A epitranscriptome of the mouse feral bran and intestine, probably by altering the expression of m^6^A writers and erasers, implying m^6^A might serve as a critical regulator for mediating the impact of microbiome to development and disease.

Besides development, RNA modifications have been reported to exert critical roles in a variety of physiological processes. With this regard, Wilkinson et al. summarized the functions and regulation of RNA modifications (e.g., m^6^A, m^5^C and m^1^A) in cellular processes, emphasizing the context-specific roles of RNA modifications during pathogenesis and the recent advances in disease prevention and therapy by targeting RNA modification.

## The functions and mechanisms of RNA modifications during pathogenesis

The aberrant regulation and function of RNA modifications is pervasively found in human diseases, particularly in cancer. Huang et al. found m^6^A demethylase ALKBH5 serves as independent favorable prognostic marker and plays tumor suppressive function by modulating iron metabolism and epithelial-mesenchymal transition (EMT) in pancreatic ductal adenocarcinoma. Moreover, there are three Review articles in this Research Topic focusing on the roles of RNA modifications in cancer. Lu et al. summarized the interaction network of non-coding RNAs (ncRNAs) and their relationship with m^6^A modification in colorectal cancer (CRC). Gupta et al. focused on the functions of tRNAs, tRNA-derived stress-induced RNAs (tiRNAs) and tRNA-derived fragments (tRFs), as well as their modifications, during tumor development and progression. Liu et al. reviewed recent findings of several common RNA modifications on mRNAs, rRNAs and tRNAs and their regulators in breast cancer.

Besides, RNA modifications emerge as key players in other chronic disease and injury. Yu et al. reported FTO, another m^6^A demethylase, played a role in hyperlipidemia-induced cardiomyopathy. A novel compound, known as LuHui Derivative, could inhibit FTO and alleviate the inflammatory response and injury in hyperlipidemia-induced cardiomyopathy. Fan et al. revealed the involvement of m^6^A methylation and its regulator in the development of liver fibrosis, a chronic liver injury that may lead to cirrhosis and even liver cancer, through performing m^6^A-seq and RNA-seq in liver fibrosis mice. Zhao et al. analyzed the expression of m^6^A regulators in the glomeruli in lupus nephritis compared with tubulointerstitium and whole kidney tissue, and established an m^6^A regulator signature that can distinguish lupus nephritis and healthy individuals. Nie et al. found m^6^A regulators displayed extensive differential expression in the cohorts of inflammatory bowel disease, in which two clusters of consensus clustering exhibit different immune phenotypes and clinical characteristics. These research provide insights that m^6^A methylation may be associated with the occurrence of these diseases; however, further studies are needed to determine its role.

## The application of RNA modifications in translational medicine

It has been anticipated that RNA modifications, similar to DNA methylation, could serve as biomarkers for clinical diagnostics. The integrative analyses by Xu et al. and Gu et al. suggested that m^6^A regulators may represent promising biomarkers for prediction of prognosis and clinical responses to targeted or immune therapy of low-grade glioma and HCC patients. Katanski et al. performed multiplex small RNA sequencing (MSR-seq) on residual nasopharyngeal swabs to test the idea of utilizing host tRNA properties as biomarkers for the clinical outcome of SARS-CoV-2. They reported that combining tRNA modifications with full-length tRNA abundance and tRNA fragmentation could provide strong power for the accurate prediction of SARS-CoV-2 infection symposium severity, shedding light on the application of tRNA modification and also potentially other RNA modifications as diagnostic biomarkers.

In clinical practice, RNA modification has become a powerful tool in making mRNA vaccines. Morais et al. reviewed the effect and mechanism of N1-methyl-pseudouridine in the successful invention of mRNA vaccines against SARS-CoV-2. From a broader view, Liu et al. summarized the roles of cap and tail modifications, nucleoside substitutes, and chimeric mRNAs on tuning the properties of mRNAs and discussed the potential of harnessing the efficacy of mRNA drugs through such mRNA modifications.

## Conclusion

The studies published in this Research Topic provide a window into the basic and translational research of RNA modifications. We hope the studies and insight provided by the research and review articles in this Research Topic could inspire researchers and bring critical thinking on the field of epitranscriptomics.
